# Sequential Self-Folding Structures by 3D Printed Digital Shape Memory Polymers

**DOI:** 10.1038/srep13616

**Published:** 2015-09-08

**Authors:** Yiqi Mao, Kai Yu, Michael S. Isakov, Jiangtao Wu, Martin L. Dunn, H. Jerry Qi

**Affiliations:** 1 The George W. Woodruff School of Mechanical Engineering, Georgia Institute of Technology, Atlanta, GA 30332, USA; 2 Singapore University of Technology and Design, Singapore

## Abstract

Folding is ubiquitous in nature with examples ranging from the formation of cellular components to winged insects. It finds technological applications including packaging of solar cells and space structures, deployable biomedical devices, and self-assembling robots and airbags. Here we demonstrate sequential self-folding structures realized by thermal activation of spatially-variable patterns that are 3D printed with digital shape memory polymers, which are digital materials with different shape memory behaviors. The time-dependent behavior of each polymer allows the temporal sequencing of activation when the structure is subjected to a uniform temperature. This is demonstrated via a series of 3D printed structures that respond rapidly to a thermal stimulus, and self-fold to specified shapes in controlled shape changing sequences. Measurements of the spatial and temporal nature of self-folding structures are in good agreement with the companion finite element simulations. A simplified reduced-order model is also developed to rapidly and accurately describe the self-folding physics. An important aspect of self-folding is the management of self-collisions, where different portions of the folding structure contact and then block further folding. A metric is developed to predict collisions and is used together with the reduced-order model to design self-folding structures that lock themselves into stable desired configurations.

Folding and unfolding are one of the most important mechanisms for generating large deformation and motions in nature, with a plethora of examples such as the winged insects^1^ and tree leaves^2^,^3^,^4^. In recent years, folding has attracted increasing interest for technological applications. A contemporary example is origami^5^, the ancient art of paper folding that has found increasing engineering applications ranging from space exploration^6^, foldable photovoltaics^7^ and batteries^8^, to shopping bags^9^,^10^, biomedical devices^11^,^12^,^13^, and metamaterials^14^. Complimentary theoretical work shows that the controlled folding and unfolding are complicated due to the independent folding motions of individual folds. Self-folding is more attractive in many applications as it saves the expensive infrastructure investment, such as robotic arms, to automate the folding^10^. Several self-folding mechanisms have been developed, such as using residual stress^15^,^16^,^17^, shape memory alloy actuation^18^, DNA^19^ and hydrogels^20^. More recently, 3D printing technology is used to print active structures, leading to the emergence of 4D printing^21^,^22^,^23^,^24^,^25^. These works demonstrated structures and machines that are modular, self-assembled, self-reconfigurable, and capable of adapting to their environments.

Shape memory polymers (SMPs) are smart materials that can recover their permanent shapes from one (or sometimes multiple) programmed temporary shape(s) when an appropriate stimulus is applied, such as temperature^26^,^27^,^28^,^29^,^30^, magnetic fields^31^,^32^,^33^, and light^34^,^35^,^36^,^37^. Since this type of smart materials has the potential to sense environmental changes and react accordingly in a predetermined sequence, they are promising candidates for spontaneous configuration-changing applications. Besides, they can be chemically tuned to achieve biocompatibility and biodegradability, and hence have been studied extensively for biomedical and aerospace applications^38^,^39^,^40^,^41^. Recently, shape memory polymers have been used to self-fold flat sheets into target structures. Dickey and co-workers^42^,^43^ demonstrated self-folding of a SMP sheet activated by unfocused light where photo-absorbing inks were printed on the SMP surface to convert light energy into heat. Felton *et al.*^44^ further developed this concept and developed self-folding robots and structures by using similar SMPs. Our group^21^,^22^ applied 4D printing technology to design and fabricate *active origami* where a flat printed sheet can be folded into a 3D structure. There, shape memory polymer fibers were precisely printed into an elastomeric matrix to create an active composite that was used to fabricate integrated hinges to enable origami folding structures. Generally, the above mentioned self-folding origami structures have relatively simple folding patterns so that different folding parts would not collide with each other, rendering the folding kinetics inconsequential in terms of realization of the desired final configuration. However, this is not generally the case. One can easily envision what appear to be simple self-folding structures that cannot actually fold themselves (as we will show in this work) because the final configuration depends on the sequence of folding of the individual pieces due to self-collisions and contact that can occur. In the recent exciting work by Felton *et al.*^45^,^46^, folding patterns were achieved by using a computer to control the activation of a sequence of heaters that are attached to individual hinges; essentially this provides a time-dependent, spatially nonuniform temperature profile to actuate the SMP hinges in a prescribed sequence.

In this work we propose an alternative and in some ways simpler method to create sequentially self-folding structures by 3D printing hinges with digital SMPs into components. We avoid the use of complicated localized heating systems and actuate the sequenced folding with a spatially uniform temperature field. The proposed method involves 3D printing hinges from digital SMP materials that can self-fold at prescribed speeds, which are controlled by the thermomechanical properties of the individual SMPs and a prescribed thermomechanical training protocol. The so-called digital SMPs are digital materials that are formed by mixing two base materials at specific ratios on a digital voxelized domain to achieve prescribed thermomechanical and shape memory behaviors. We demonstrate the concept by designing and fabricating a series of complex self-folding structures and showing how we can control the folding by using SMPs with a uniform spatial temperature. A reduced-order model (ROM) is also developed with a metric to predict self-collisions of folding parts to simulate the folding process. We show this model is in good agreement with experiments and companion high-fidelity finite element simulations, and hence can be used to design self-folding and self-locking structures.

## Results

### Hinge Folding

**Figure 1:**
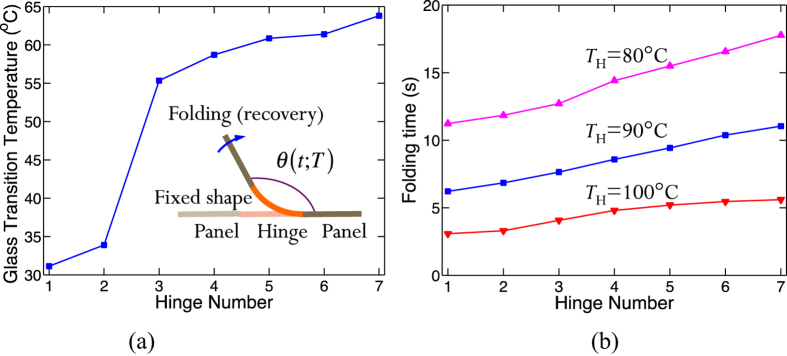
(**a**) Glass transition temperatures of the seven digital SMPs used for hinge materials; the inset shows a schematic view of the folding of an SMP hinge; (**b**) folding time of hinges to obtain 98% recovery ratio at various recovery temperatures.

Hinges designed with digital SMPs with specified thermomechanical behaviors, especially recovery, are the key element of sequentially self-folding structures (shown in the inset of Fig. 1a). We design, fabricate, and test hinges using seven different digital SMPs. We use H-i (i = 1, 2, 3 …7) to represent the *i*-th hinge material and the hinges all have a thickness of 0.8 mm and a width of 6 mm. The glass transition temperatures (*T*_g_s, see the Method for details) of these hinge materials are shown in Fig. 1a. The shape memory behavior of the hinges, including shape fixity and shape recovery, is controlled by the applied mechanical and thermal-temporal programming and recovery conditions. However, different programming methods can lead to the same recovery profile and fixity^47^ and we exploit this in the design of hinges. To understand the basic hinge behavior we consider a hinge printed with an initial angle of *θ* = 90° and then programmed (by bending at *T*_H_ = 90 °C) to assume a temporary flat state (*θ* = 180°), which corresponds to a shape fixity of 100%. Then we recover the hinges to their original shape (*θ* = 90°) by heating them in a constant temperature bath, during which we monitor the recovery of the hinges as a function of time. We quantify the shape recovery with the angular recovery ratio (*R*_*r*_):


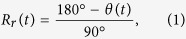


where *θ(t)* is the time dependent angle (Fig. 1a).

The time-dependent angular recovery of the bending hinges can be examined by using a simple scaling approach with respect to the uniaxial shape memory behavior, which is modeled by a multi-branch constitutive model^48^,^49^,^50^ with 16 branches. Details regarding the model and its parameter identification can be found in the Supplementary Materials along with a demonstration of how it can be used to describe the rotational behavior of hinges during bending. Figure 1b shows the folding time at three different folding temperatures for the seven hinges by the 1D model. Here, the folding time is defined as the time needed for a hinge to fold to *θ* = 91.8°, which corresponds to a recovery ratio of 98%. It can be seen that at the same folding temperature, hinges fabricated by higher *T*_*g*_ materials (the higher hinge numbers) need longer times to fold. In addition, the differences of folding times among these hinges increase as the temperature decreases. For example, if 80 °C is used as the recovery temperature, the difference between H-1 and H-7 is about 7 seconds; but at 100 °C, the difference becomes about 2 seconds. The differences in the folding times provide the opportunity to design the sequencing of folding, as will be shown next.

### Sequential self-folding

**Figure 2:**
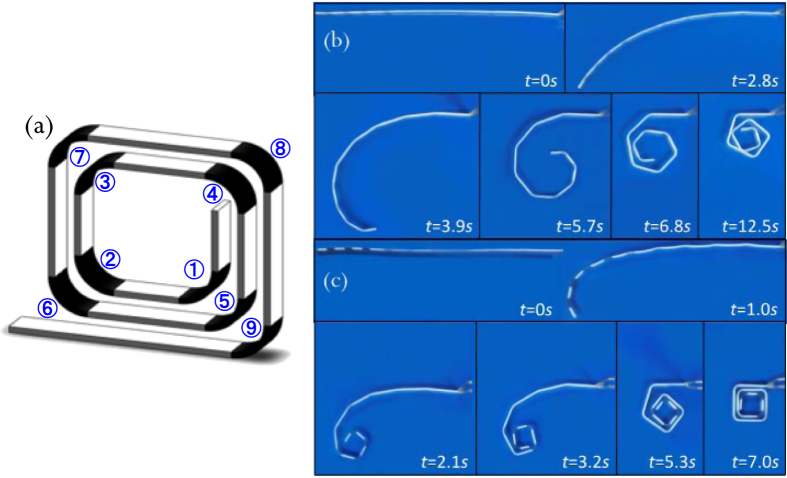
(**a**) The schematic graph of the helical SMP component. Series of photographs showing the shape recovery process of the helical SMP component (**a**) with uniform hinge sections, and (**c**) with graded hinge sections.

We design, fabricate by digital SMP printing, and test a seemingly-simple helical structure where rigid (non-active) panels are connected by active hinges on each corner with a radius of 5 mm (Fig. 2a). The thickness of the panels and hinges is 0.8 mm, the depth is 6 mm and the clearance between neighboring panels is 5 mm. We program the self-folding structure by unfolding it into flat at an elevated programming temperature, which results in each of the hinges bending to the prescribed curvature. Here the prescribed curvature is achieved by a simple state of deformation (bending) and is amenable to analysis, but in general more complex deformations can be programmed. We then recover the desired final configuration by heating the structure uniformly to *T*_r_. To demonstrate the importance of the folding dynamics and self-collisions in the process we design two structures that have identical geometry, but each has a different set of materials used for the hinges. In Sample 1 each of the 9 hinges uses the same SMP material (H-3), and in Sample 2 the hinges are made from seven different materials (H1-H7; Table 1). After printing the structure we deform it into a flat configuration in hot water at *T*_H_ = 90 °C, which is above the *T*_g_ of all of the SMP sections. Then the sample is cooled to *T*_L_ = 10 °C, at which all the SMP hinges are in their glassy states. After releasing the external load, the structure is fixed at the temporary straight shape. To activate the shape recovery of the structure, we immerse it in hot water with *T*_r_ = 90 °C so that all hinges experience the same thermal conditions. The shape recovery process is monitored by a video camera.

Figure 2b shows the recovery sequence of Sample 1. Since all the hinges possess the same thermomechanical behavior (H_3_ with *T*_*g*_ = ~55 °C), they start to recover their shape simultaneously. Before the inner hinges can fully fold, the outer layers of the sample coil back at the same speed. The uncoordinated and simultaneous motions of all hinges lead to two undesirable results. First, at ~7.5 s, parts of the structure collide with each other because of folding pathway interference. The contact between different parts provides frictional resistance and leads to a significant reduction of folding speed. As seen in the figure, from 7.5 s to 12.5 s, folding proceeds slowly. Second, at 12.5 s, the frictional force becomes so large that folding stops and the structure fails to reach its final target shape.

For the structure shown in Fig. 2a, in order to recover its final target shape, the inner hinges require faster folding speeds than those of the outer hinges, which can be accomplished by assigning SMPs with lower *T*_*g*_s. *T*o this end we designed hinges unique to each corner as (shown as Case 2 in Table 1): H-1 with the lowest *T*_*g*_ (~32 °C) is assigned to the two innermost corners; H-7 with highest *T*_*g*_ (~65 °C) is assigned to the two outermost corners; the rest corners are assigned with hinges H-2 to H-6 with gradually increased *T*_*g*_.

After experiencing the same programming procedure as described in the previous section, the structure is immersed in hot water at *T*_H_ = 90 °C and the free recovery process is monitored. Figure 2c shows the snapshots of the material configurations at different recovery times, which visually demonstrates the sequential recovery of hinges to yield unconstrained and rapid (~7.0 s) folding to the target configuration as shown in Fig. 2c. The different folding speeds of the hinge sections due to their respective *T*_*g*_s enable the avoidance of self-collisions. Since the folding speeds of the three inner hinges are faster than the outside ones, they first exhibit shape recovery (they start the shape recovery within ~1 s and finish in ~3 s). Then shape change is successively triggered along the helical line and the last two hinges start to recover after 3 s and finish at ~7.0 s. The hierarchical shape recovery profiles of the hinge sections enables the successful sequential shape recovery in the SMP component without interruption by collisions.

The above two cases demonstrate the importance of design and the ability to control the assembly sequence and dynamics by the spatial arrangement of SMPs with varying thermomechanical behavior. A “bad” design without careful coordination of different folding panels can lead to slower and even failed folding of the entire structure.

### Folding simulations: finite element analysis and reduced-order model

To describe both unfolding and folding behaviors and use this understanding in the current design, we take a two-stage approach for analysis. First, we simulate the folding dynamics using finite element analysis (FEA). FEA provides high-fidelity simulation of the spatial and temporal variation of the programming (unfolding) and deployment (folding) processes as it accurately takes into account the complicated thermomechanical constitutive behavior of individual hinges as well as their interactions with the stiff, non-deforming panels. However, it is also time-consuming as it involves complicated contact (used for unfolding) as well as nonlinear geometrical mechanics. Since the panels are relatively rigid compared to the hinges and the focus of our work is the sequential folding and the potential issue of collisions during folding, we also develop a reduced-order model (ROM) to simulate rigid body motions of the panels (driven by the recovery of angular shape recovery of hinges) to investigate the folding pathways. During folding and unfolding the deformation is mainly localized in the hinges so in the ROM we idealize the structure as rigid panels connected by hinges with rotational behavior given by our constitutive model as described in Methods and Supplementary Materials sections. The simplicity offered by the ROM also facilitates the development of a simple criterion to predict if collisions will occur during self-folding before a structure is printed.

**Figure 3:**
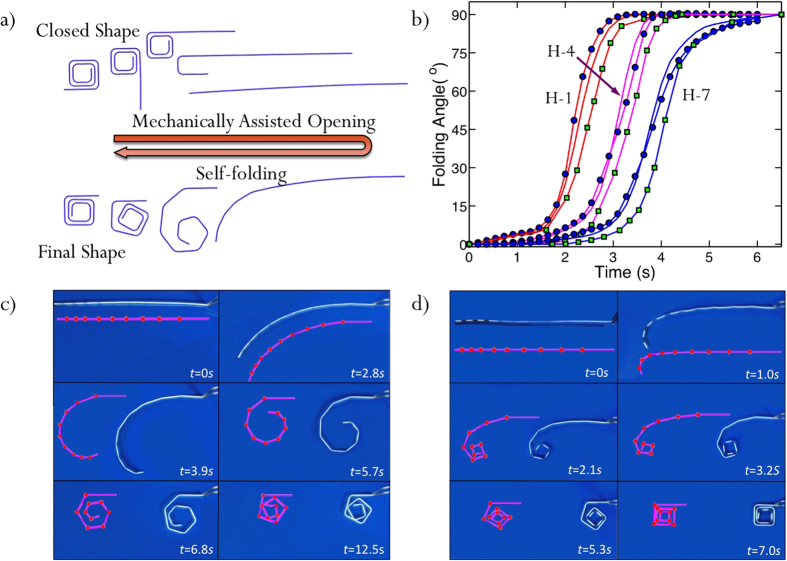
(**a**) The Finite element simulation of shape memory recovery activated sequential self-folding after the strip is firstly programmed at high temperature. (**b**) The simulations of folding angles of three representative hinges by ROM and compared with those by FEA and experiments. Solid-line only: FEA; round dot: ROM; square: experiments. Red color: H-1; magenta color: H-4; Blue color: H-7. Comparison between ROM simulations and experiments for (**c**) Sample 1 and (**d**) Sample 2, where red lines indicate the simulation results.

Figure 3a shows finite element simulations of the folding and unfolding processes. In the unfolding process, the structure is opened by rigid surfaces to successively unfold one panel after the other at a high temperature. After lowering the temperature and unloading, the structure is fixed into the flat configuration. Recovery is achieved by heating the structure uniformly and it folds into the original configuration as hinges are sequentially activated due to their individual time-dependent constitutive response. The simulated recovery ratios for representative hinges (H-1, H-4, and H-7) are presented in Fig. 3b along with measurements and results form the ROM. The FEA predictions are in good agreement with the measurements, although they predict slightly faster folding than the experiment, probably due to the resistance from the water in the experiment, which is neglected in the simulation. Figure 3b also shows the ROM simulations and they are in good agreement with the FEA simulations and experiments. Figure 3c,d show ROM simulations of the two samples in Table 1. They describe both the unsuccessful (Fig. 3c) and successful (Fig. 3d) folding sequences well. Importantly the ROM captures the self-collisions that occur during the failed folding; based on this capability, we use it to design more complex folding scenarios in the next section.

### Collision determination

**Figure 4:**
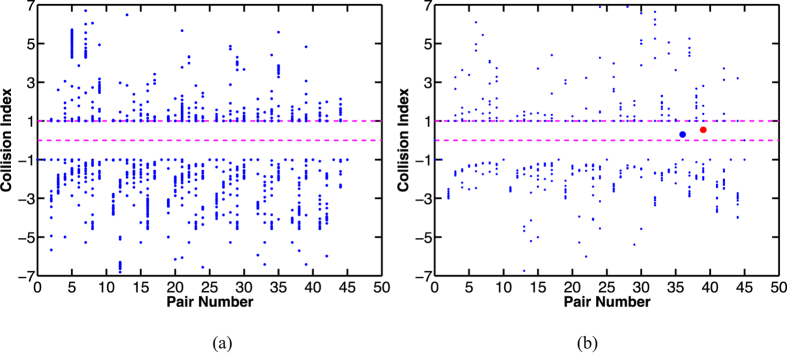
The self sequential self-folding (a) without collisions; (b) with collisions. When there is no collision, all collision indices are out of span (0, 1) (shown in (**a**)), and when there is a collision, the index falls in span (0, 1) (as shown in (**b**), two points (a red and a blue point) are in span (0, 1)). The red dot between (0, 1) corresponds to first collision; the blue dot corresponds to the second collision.

Utilizing the ROM simulation, we develop a simple *collision index*, which can be used to determine if two panels will collide during the folding process. As described in the Methods section, the collision index, *S*_*ij*_, is calculated based on the motion of any pair of panels (the *i*-th panel and the *j*-th panel) during each time increment of the simulation. In general, when 0 < *S*_*ij*_ < 1, a collision will occur. Figure 4a,b show the collision indices of the two designs discussed above; there are 10 panels in each design which gives rise to 45 unique panel pairs. In Fig. 4a, the structure can fold without collisions, therefore, no collision index falls between the band between 0 and 1 during the simulation. In Fig. 3c two collisions occurred between panels and the structure cannot fold to its target shape; Fig. 4b reflects this as two collision indices fall in span(0, 1). In the figure, the red dot between (0, 1) corresponds to first collision; the blue dot corresponds to the second collision. More collision will happen; but since our interest is the first collision, no further calculation is conducted.

### A self-locking system

**Figure 5:**
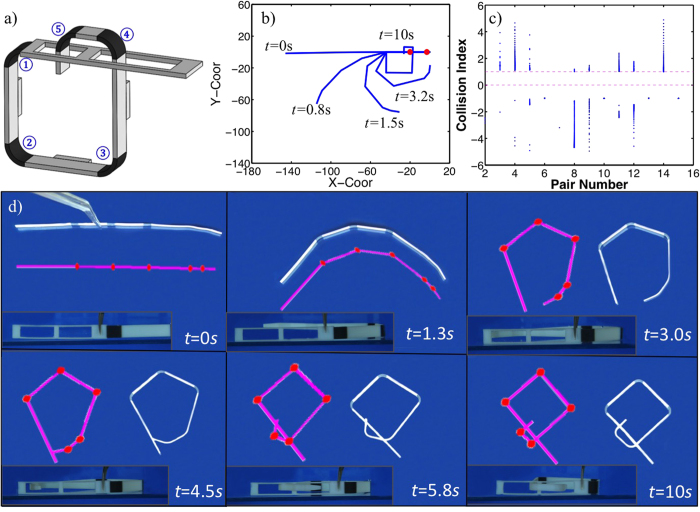
(**a**) The schematic graph of the interlocking SMP component. (**b**) The ROM simulation of the sequential folding resulting in interlocking, and (**c**) the collision indices of the chosen design. (**d**) The comparison of experiments (plan and side views) and the ROM simulations (red lines).

Here we use our ROM to design and then 3D print a self-folding and self-locking structure, as shown in Fig. 5a. While this locking structure demonstrates the importance of controlling the folding sequence and how more complex structure folding can be obtained, we could also pursue other kinematic objectives such as making stops, kickstands, etc. Here, two holes with different dimensions are designed on one of the end panels. The objective is to sequentially guide the other end of the structure through the first hole and then through the second hole to lock the structure in place. The thickness of the SMP components is 0.6 mm (0.8 mm for the plates with holes) and the depth is 6 mm. Five hinges (with a radius of 5 mm in uniform) are used.

In order to ensure that the end of the strip passes through the two holes in sequence, we design each hinge to control the folding rate of each segment of the structure. This results in two requirements: First, the folding of hinges 1 and 2 should be faster than the others so that the two end sides reach each other in the correct position and angle. Second, folding of hinges 4 and 5 should be slower to guarantee them to pass through the two holes successively and then lock the structure. Using our ROM simulations with the collision index, we determined feasible folding rates for each hinge, and then realized them with hinges from Fig. 1. Specifically we assigned hinges H-2, H-2, H-3, H-6 and H-6 for hinges 1 to 5 in Fig. 5a.

The self-folding and self-locking structure is demonstrated experimentally in Fig. 5. The structure shown in Fig. 5a is first printed. At the temperature of 90 °C, it is opened into a flat strip, or namely the unlocked configuration. It is then cooled to 10 °C where the flat shape is maintained. After this programming step, the unlocked structure is immersed in hot water (90 °C) and the free recovery process is monitored from both the top and side, as shown in the snapshots of Fig. 5d. Fig. 5b,c show the ROM simulated folding and locking procedure. As predicted by ROM, the active locking side is able to pass through the two holes successively, and finally recovers to the initial configuration. The ROM simulation and the experiment show good agreement.

### A 3D folding structure

**Figure 6:**
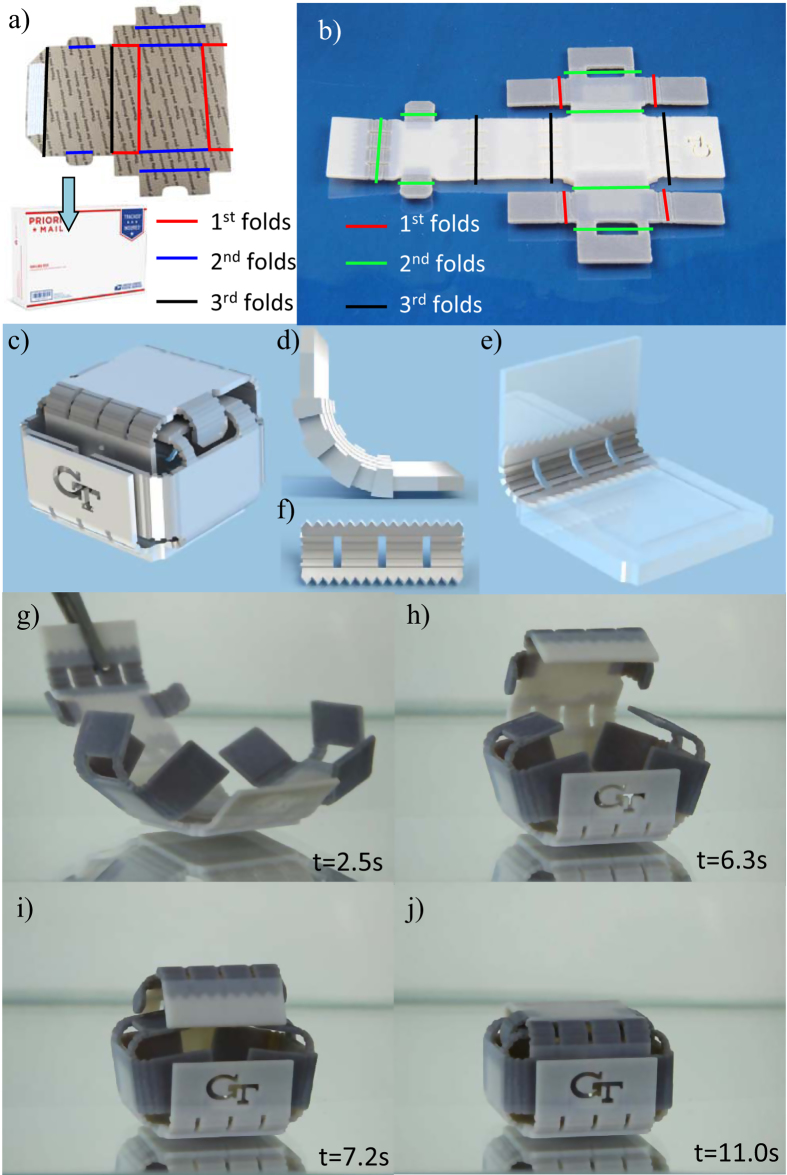
3D folding structures mimicking the USPS mailbox. (**a**) A USPS mailbox is folded into a box by following a sequence of folding. (**b**) A programmed 3D printed sheet with different materials assigned at different hinges. (**c**–**f**) The design of the folding box with some details at the hinges. (**g**–**j**) Upon heating, the sheet folds into a box with self-locking mechanism.

Folding boxes are notable folding structures in daily life and widely used in packaging. They normally require a folding sequence in order to form a stable and fully packed structure. Taking a USPS mailbox (Fig. 6a) as an example, one can have a sheet of hard board paper cut into a specific shape. At the time of usage, one folds the box in a sequential manner; the red lines represent the first group of folds, where some lips are folded to form supports; the blue lines represents the second group of folds to partially close the box; the final folds (black lines) insert the two small lids (on the left top and bottom) into the holes formed by the long blue line folds to increase the stability of the box; also the white color patch represents glue that will seal the structure to from a final strong and stable box. Inspired by this, we design a 3D folding box with an internal locking mechanism, as shown in Fig. 6b. Three types of hinges are used. Hinge H-3 is used on the smaller lips to enable fast folding of these lips (red lines). Hinge H-5 is used in the location indicated by the green line, to enable intermediate folding speeds such that some self-support and self-locking mechanism can be formed. Hinge H-6 (black lines) has the slowest folding speed and is used to form the final structure. The original shape of the box is shown in Fig 6c. Since curved portions of the hinges are not in the printing plane, these hinges are suspect to damage during unfolding. To improve the damage tolerance, hinges are specially designed (Fig. 6d–f). A periodic step profile is employed along the surface contour of the hinge (Fig. 6d). Thinner stepwise sections require lower bending stress and have higher thermal conductivity, making it both easier and faster to unfold complex assemblies. Additionally, the periodic profile allows for a wider range of bending motion since the hinge is not subject to restrictive compressive strains. Thicker stepwise sections serve to maintain part rigidity through higher average modulus than a thin hinge alone. To secure the interface between hinges and structural faces, the hinges are embedded within the structural faces and reinforced with a triangular interface to maximize axial strength (Fig. 6e). Longer hinges are equipped with slots cut along the length of the jagged contours to isolate fracture points such that the assembly would not be compromised should a fracture occur (Fig. 6f). Figure 6g–j show the folding sequence. The whole structure folds back in about 11 seconds.

## Discussion

Self-folding structures are an important contemporary research area that has great potential to impact a variety of technological applications, such as biomedical devices, soft robots, and space structures. In most previous work, self-folding was achieved by spatially controlled heating zones, which are generally difficult to fabricate. In this paper, we develop a new approach that results in easier manufacture of self-folding structures by 3D printing multimaterial integrated structures with hinges made of digital shape memory polymers with different thermomechanical properties at spatially-varying locations to facilitate self-folding by uniformly heating the structure after the thermomechanical programming step. Most previous efforts to self-folding have also focused on folded shapes that were kinematically simple in that the folded shape could be achieved without consideration of the sequence of folds. Even for fairly simple folded shapes as we considered here, we show folding path planning is critical for the successful completion of folding sequence, and this requires careful design of folding hinges. To this end we develop both high-fidelity finite element analysis simulations and a reduced-order model to describe the folding process. Although finite element simulations provide more comprehensive understanding of the folding procedure, it is time-consuming. The reduced-order model, which is verified by finite element simulations, considers the folding as rigid body motions of panels, whose motions are driven by the rotation of hinges with prescribed time-dependent constitutive response, and is able to capture the folding accurately and efficiently. When coupled with the collision index, it is an effective tool for the design of self-folding structures with complex kinematics, including self-locking and USPS mailbox.

There are some limitations of the current work. First, the shape memory effects in the 3D printed material can only have one-way actuation, i.e., if a second actuation is desirable, one has to conduct a second programming. The development of two-way actuation design is currently undertaken and will be reported in the near future. Second, folding time can be improved by using a higher recovery temperature. However, there is a limit on how fast an SMP can recovery, which we showed in our previous work to be about a few seconds^49^,^51^, as the total recovery time is determined by the times for heat transfer and material intrinsic recovery. Third, the printed materials tend to break easily, limiting the available reliable 3D structures. Therefore, it is desirable to have printed materials with good fracture toughness.

The development of practical and versatile digital SMP solids will enable self-adjusting and self-reinforcing structures to cope uniquely with different environmental conditions. It should be noted 3D printing is not the only method to create materials/structures with functional gradient. For example, functional graded SMPs were demonstrated before^52^. However, by using multimaterial 3D printing technology, self-folding structures using digital SMPs with both spontaneous and sequential folding behavior can be directly created with large design freedom and high resolution of spatial material properties distribution in complicated 3D structures. Besides, the manufacturing route can be easily implemented with the potential for immediate engineering applications for low-cost and rapid production. With the emergence of voxel printing, we believe the concept and examples demonstrated in this paper can be used for developing future self-deployable structure with more complicated folding patterns.

## Methods

### Materials and experiments

The materials used in this study are digital materials that are available from the multimaterial 3D printer (Objet Connex 260, Stratasys, Edina, MN, USA). The so-called digital materials refer to those created through mixing two base model materials, i.e., VeroWhite and Tangoblack, by jet spraying before UV curing. VeroWhite is a rigid plastic at room temperature polymerized with ink containing isobornyl acrylate, acrylic monomer, urethane acrylate, epoxy acrylate, acrylic monomer, acrylic oligomer, and photo-initiators. TangoBlack is a rubbery material at room temperature polymerized by monomers containing urethane acrylate oligomer, Exo-1,7,7-trimethylbicyclo [2.2.1] hept-2-yl acrylate, methacrylate oligomer, polyurethane resin and photo-initiators. The digital materials consist of varying compositions of these two materials that lead to different thermomechanical properties. In this work, the thermomechanical properties of seven digital materials (respectively labeled as polymer 1-7) are tested. After all the samples are printed with the dimension of 15 mm (height) × 3 mm(width) × 0.6 mm(thickness), thermomechanical properties of these polymers are characterized by a dynamic mechanical analyzer (DMA, Model Q800, TA Instruments, New Castle, DE, USA). The samples are firstly heated to 100 °C in the DMA tester and stabilized for 20 minutes to ensure thermal equilibrium, then a preload of 1 KPa is applied. During the DMA test, the strain is oscillated at a frequency of 1 Hz with a peak-to-peak amplitude of 0.1% while the temperature is decreased from 100 °C to a prescribed low temperature at a rate of 2 °C/min. The temperature corresponding to the peak of the tan δ curve is taken to be the glass transition temperature *T*_*g*_ 53,54 (Fig. S2).

### Finite element analysis simulations

FEA simulations of unfolding and folding as shown in Fig. 3 are conducted using the FEA software ABAQUS (Simulia, Providence, RI, USA) with a user-defined constitutive model implemented in a UMAT subroutine. The fitted material parameters are imported into the 3D finite deformation constitutive model. To simplify the FEA simulation, a 2D plane strain model is used. The hybrid CPE4HT element in ABAQUS element library is used, and hinge assignments are shown as case 2 in Table 1 while the non-active panels are considered as a linear elastic material with high modulus of 1GPa, which is much higher than the modulus of the SMPs in their rubbery state. For the FEA simulation, the strip is firstly unfolded into a straight configuration at 90 °C, which is above T_g_ of all the SMP material sections, as shown as the process in Fig. 3a by using rigid surfaces to unfold one panel after the other, which is consistent with the prescribed deformation of the experiments. Once the whole structure is deformed into a flat plate, it is cooled to 10 °C, where all the hinge sections are in their glassy states. The external loading and constraints are then removed and the temporary configuration is fixed. The structure is then uniformly heated to a high temperature, causing the structure to fold sequentially.

### Reduced-order model simulations

**Figure 7:**
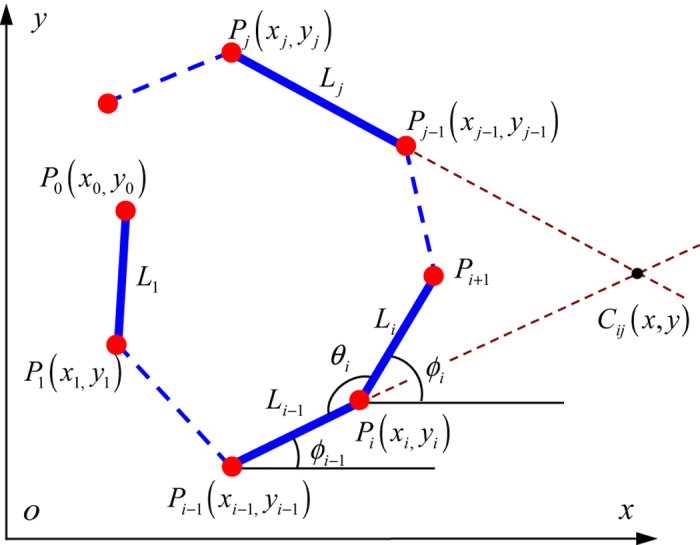
Schematic of two arbitrary strips having a factitious intersection point.

We use the scaling rule to predict the shape recovery ratio of hinge by the recovery behavior of a sample under linear stretch. Here, we assume that the folding structure can be represented by rigid panels connected by hinges, which occupy infinitesimal space but control the angles between two panels. Figure 7 shows panels are connected by hinges, which are represented by *P*_i_ with its coordinates of (*x*_i_, *y*_i_) for the i-th hinge.

In the ROM, we assume that the angular rotation of the hinge is equal to the linear recovery ratio of a SMP. This assumption is validated in the Supplementary Material. Following Eq. 1, we have


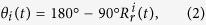


where 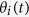 is the angle between the *i*-th panel and (*i* + 1)-th panel, 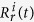 is the recovery ratio of the *i*-th hinge, which is between the *i*-th and (*i* + 1)-th panels. In order to calculate the location of the hinges, the orientation of the i-th panel is calculated as


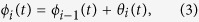


where 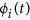 is the orientation of *i*-th panel. Finally, the coordinates of the *i*-th hinges are


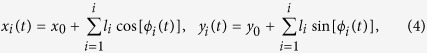


where 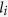 is the length of the *i*-th panel.

### Collision index

We consider two arbitrary panels in the structure (as shown in Fig. 6), denoted as *L*_*i*_, *L*_*j*_, with coordinates of points as 
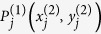
, 
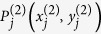
, respectively. The parametric equation for the *i*-th panel (denoted as a solid line) is given as


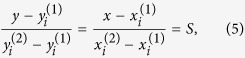


where *S* is a parameter that is the distance from point *i* to point (*x*, *y*) normalized by the length of the panel. Therefore, for the point (*x*, *y*) on the panel, 0 < *S* < 1. By using the parametric equations of the *i*-th panel and the *j*-th panel, the coordinates of the intersection between these two panels can be calculated as *C*_*ij*_(*x*_*ij*_, *y*_*ij*_). Substituting the coordinate of intersection point (*C*_*ij*_(*x*_*ij*_, *y*_*ij*_)) into the *i*-th line equation (Eq. (4)) yields a parametric index *S*_*i*_. When 0 ≤ *S*_*i*_ ≤ 1, the intersection point is on the *i*-th panel, while when *S*_*i*_ > 1 or *S*_*i*_ < 0 the intersection point sits on the extension line of the *i*-th panel. The same is true for parametric index, *S*_*j*_, for the *j*-th panel. It is thus easy to conclude that when the two panels intersect, we have 0 ≤ *S*_*i*_ ≤ 1 and 0 ≤ *S*_*j*_ ≤ 1; otherwise there is no intersection. We further define an interaction index for intersection of line *i* and line *j* as


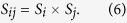


In general, when 0 < *S*_*ij*_ < 1, intersection occurs. However, three cases that can give 0 < *S*_*ij*_ < 1 but no intersection: (1) 0 < *S*_*i*_ < 1 and *S*_*j*_ > 1 (for example, *S*_*j*_ is slightly larger than 1); (2) 0 < *S*_*j*_ < 1 and *S*_*i*_ > 1 (for example, *S*_*i*_ is slightly larger than 1); (3) *S*_*i*_ < 0 and *S* _*j*_ < 0.

In ROM simulations, at each time increment, the coordinates of individual hinge points are first calculated; the intersections points and the intersection indices are calculated. If 0 < *S*_*ij*_ < 1, the above three conditions will be checked; if any one of them is satisfied, there is no intersection; otherwise, an intersection exits and folding cannot be completed.

## Additional Information

**How to cite this article**: Mao, Y. *et al.* Sequential Self-Folding Structures by 3D Printed Digital Shape Memory Polymers. *Sci. Rep.* **5**, 13616; doi: 10.1038/srep13616 (2015).

## Supplementary Material

Supplementary Information

## Figures and Tables

**Table 1:** Hinge assignment of each fold.

Fold Number		1	2	3	4	5	6	7	8	9
Material	Sample 1	H-3	H-3	H-3	H-3	H-3	H-3	H-3	H-3	H-3
	Sample 2	H-1	H-1	H-2	H-3	H-4	H-5	H-6	H-7	H-7
