# Supplementary material for: Sequential Self-Folding Structures by 3D Printed Digital Shape Memory Polymers
Source: Sci Rep. 2015 Sep 8;5:13616. doi: 10.1038/srep13616 (PMC4562068; doi:10.1038/srep13616)
Supplement: Supplementary Information [file srep13616-s1.pdf]

Supplementary Materials

Sequential Self-Folding Structures by 3D Printed Digital Shape Memory Polymers

Yiqi Mao[1], Kai Yu[1], Michael S. Isakov[1], Jiangtao Wu[1], Martin L. Dunn[2*], H. Jerry Qi[1*]

[1]The George W. Woodruff School of Mechanical Engineering
Georgia Institute of Technology
Atlanta, GA 30332, USA

[2]Singapore University of Technology and Design, Singapore

Corresponding authors: martin_dunn@sutd.edu.sg; qih@me.gatech.edu

S1. Description of Multi-branch Model

The multi-branch model (also referred as generalized standard linear solid model) has been shown to be able to capture the shape memory effects of the polymers[1]. Figure S1 shows a schematic representation of the model, where the number of branches depends on the width of glass transition temperature range and the structure of the polymers.

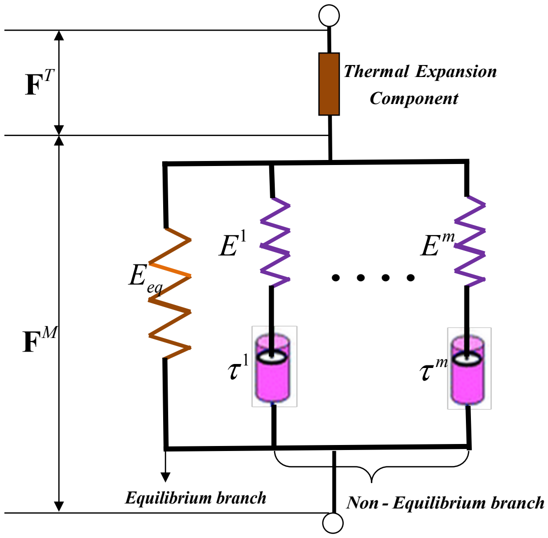

Figure S1 1D rheological representain of the multi-branch model

By applying the Boltzmann's superposition principle, the constitutive model can be rewritten as [2,3],

$$\sigma(t) = E_{eq} e_m(t) + \sum_{i=1}^{n} E_i e_i^e \,, \tag{S1}$$

with

$$e_i^e = \int_0^t \dot{e}_m(s) \exp\left(-\int_s^t \frac{\mathrm{d}t'}{\tau_i\left(T, t'\right)} \right) \mathrm{d}s \,, \tag{S2}$$

where $e_m(t)$ is the total strain, E_{eq} and E_i are the elastic moduli in the equilibrium

and nonequilibrium branches, respectively. $\tau_i(T)$ is the temperature-dependent relaxation time in the dashpot of each nonequilibrium branch and follows the well-known time-temperature superposition principles[4], i.e.,

$$\tau_i(T) = \tau_0^i \alpha_T(T), \text{ for } 1 \le i \le m, \tag{S3}$$

where $\alpha_T(T)$ is the time-temperature superposition (TTSP) shifting factor and τ_0^i is the relaxation time at the reference temperature when $\alpha_T(T)=1$. At temperatures around or above a reference temperature T_s, the WLF equation is applied,

$$\log \alpha_T(T) = -\frac{C_1\left(T-T_M\right)}{C_2+\left(T-T_M\right)}, \tag{S3}$$

where C_1 and C_2 are material constants and T_M is the WLF reference temperature. When the temperature is below T_s, $\alpha_T(T)$ follows the Arrhenius-type behavior:

$$\ln \alpha_T(T) = -\frac{AF_c}{k_b}\left(\frac{1}{T}-\frac{1}{T_g}\right). \tag{S4}$$

A finite deformation constitutive model can be developed based on the above linear viscoelastic model[5]. Eq. S1-S4 can be implemented into a Matlab code.

S2. Material parameters

All the material parameters are obtained for the seven digital materials tested in this work.. The method to calibrate corresponding material parameters for all equilibrium and non-equilibrium branches can be found in our previous work[1]. The fitted storage modulus and $\tan\delta$ are presented and compared with the experiment in Fig.S2.

Table S1. Modulus and relaxation time of nonequilibrium branch of the constitutive model

Poly.1	$E^1 \sim E^6 (MPa)$	2.1e3	2.7e2	2.1e2	2.0e2	3.2e2	1.5e2
	$\tau_0^1 \sim \tau_0^6$ (s)	2.1e-5	2.0e-4	5.5e-3	6.3e-2	3.1e-1	3.1
	$E^7 \sim E^{12} (MPa)$	6.8e1	2.8e1	1.1e1	4.9	3.0	3.e-1
	$\tau_0^7 \sim \tau_0^{12}$ (s)	2.6e1	2.2e2	2.0e3	2.0e4	2.0e5	2.3e6
	$E^9 \sim E^{16} (MPa)$	1.0	1.0	1.0e-1	1.0e-1		
	$\tau_0^9 \sim \tau_0^{16}$ (s)	1.0e8	1.0e9	1.0e10	1.0e11		
Poly.2	$E^1 \sim E^6 (MPa)$	1.8e3	2.4e2	2.8e2	2.8e2	1.5e2	8.7e1
	$\tau_0^1 \sim \tau_0^6$ (s)	2.0e-5	2.e-3	1.e-2	7.7e-2	8.9e-1	9.1
	$E^7 \sim E^{12} (MPa)$	4.8e1	2.5e1	1.3e1	5.9	2.8	1.4
	$\tau_0^7 \sim \tau_0^{12}$ (s)	84	7.2e2	5.7e3	4.6e4	3.8e5	3.5e6
	$E^9 \sim E^{16} (MPa)$	5.1e-1	1.0e-3	1.0e-4	1.0e-5		
	$\tau_0^9 \sim \tau_0^{16}$ (s)	2.0e7	1.0e9	1.e10	1.0e11		
Poly.3	$E^1 \sim E^6 (MPa)$	2.0e3	2.1e3	6.0e2	2.7e2	3.2e2	2.1e2
	$\tau_0^1 \sim \tau_0^6$ (s)	1.0e-5	1.e-4	6.0e-4	1.e-2	1.e-1	1.
	$E^7 \sim E^{12} (MPa)$	1.3e2	7.8e1	4.1e1	1.7e1	6.2	1.5
	$\tau_0^7 \sim \tau_0^{12}$ (s)	9.7	7.9e2	5.8e2	3.8e3	2.5e4	2.0e5
	$E^9 \sim E^{16} (MPa)$	1.1e-1	4.1e-3	1.0e-3	1.0e-4		
	$\tau_0^9 \sim \tau_0^{16}$ (s)	2.8e6	2.0e7	2.0e8	2.0e9		
Poly.4	$E^1 \sim E^6 (MPa)$	4.8e2	3.1e2	2.9e2	2.1e2	1.2e2	6.5e1
	$\tau_0^1 \sim \tau_0^6$ (s)	2.3e-5	4.6e-4	6.8e-3	7.1e-2	6.7e-1	5.1
	$E^7 \sim E^{12} (MPa)$	2.5e1	5.6	1.0	1.0	1.0e-1	1.e-1
	$\tau_0^7 \sim \tau_0^{12}$ (s)	34.1	2.e2	2.e3	1.0e5	1.0e6	1.e7
	$E^9 \sim E^{16} (MPa)$	1.0e-3	1.0e-4	1.0e-5	1.0e-6		
	$\tau_0^9 \sim \tau_0^{16}$ (s)	1.0e8	1.0e9	1.0e10	2.0e10		
Poly.5	$E^1 \sim E^6 (MPa)$	3.8e2	3.0e2	3.9e2	2.6e2	1.7e2	9.4e1
	$\tau_0^1 \sim \tau_0^6$ (s)	7.5e-4	6.8e-3	0.1	0.99	8.6	61.
	$E^7 \sim E^{12} (MPa)$	3.9e1	1.2e1	2.7	9.e-1	3.6e-2	1.e-2
	$\tau_0^7 \sim \tau_0^{12}$ (s)	3.9e2	2.5e3	2.e4	2.e5	2.3e6	2.0e7
	$E^9 \sim E^{16} (MPa)$	1.0e-3	1.0e-4	1.0e-4	1.0e-5		
	$\tau_0^9 \sim \tau_0^{16}$ (s)	2.0e8	2.0e9	2.0e10	2.0e11		
Poly.6	$E^1 \sim E^6 (MPa)$	2.e2	2.e2	2.7e1	2.1e1	3.2	1.5
	$\tau_0^1 \sim \tau_0^6$ (s)	1.e-4	1.e-3	1.e-2	1.e-1	1	8.2
	$E^7 \sim E^{12} (MPa)$	6.e-1	2.e-1	1.e-1	3.e-2	3.e-2	1.e-3
	$\tau_0^7 \sim \tau_0^{12}$ (s)	4.3e1	2.e2	1.e4	1.e5	1.e6	2.e6
	$E^9 \sim E^{16} (MPa)$	1.e-4	1.e-5	1.e-6	1.e-7		
	$\tau_0^9 \sim \tau_0^{16}$ (s)	2.e7	2.e8	2.e9	2.e10		
Poly.7	$E^1 \sim E^6 (MPa)$	9.e1	1.e1	2.8e2	2.6e1	1.9	1.3

$\tau_0^1 \sim \tau_0^6$ *(s)*	2.e-5	2.e-4	8.e-3	1.e-1	9.e-1	7.2
$E^7 \sim E^{12}$*(MPa)*	5.e-1	3.e-1	1.e-2	1.e-3	2.e-3	1.e-3
$\tau_0^7 \sim \tau_0^{12}$ *(s)*	3.6e1	2.e2	1.e4	9.e5	2.1e6	2.e7
$E^9 \sim E^{16}$*(MPa)*	2.7e-4	1.5e-5	1.e-6	1.1e-7		
$\tau_0^9 \sim \tau_0^{16}$ *(s)*	2.e8	3.e8	2.e9	2.e10		

Table S2. Parameters of the applied constitutive model

	E_s (MPa)	*AFKB*	*C_1*	*C_2*	*T_g (K)*	*T_m*
Poly.1	3.2	-9500	13.5	65.6	304.2	294.3
Poly.2	2.4	-9800	14.1	52.8	306.7	288.1
Poly.3	5.2	-10000	12.6	47.6	327.6	308.5
Poly.4	8.2	-21800	8.6	51.6	331.3	320.3
Poly.5	8.9	-10800	11.6	52.6	332.7	315.3
Poly.6	9.1	-10000	7.3	53.6	345.7	327
Poly.7	9.0	-15000	7.1	55.8	346	328

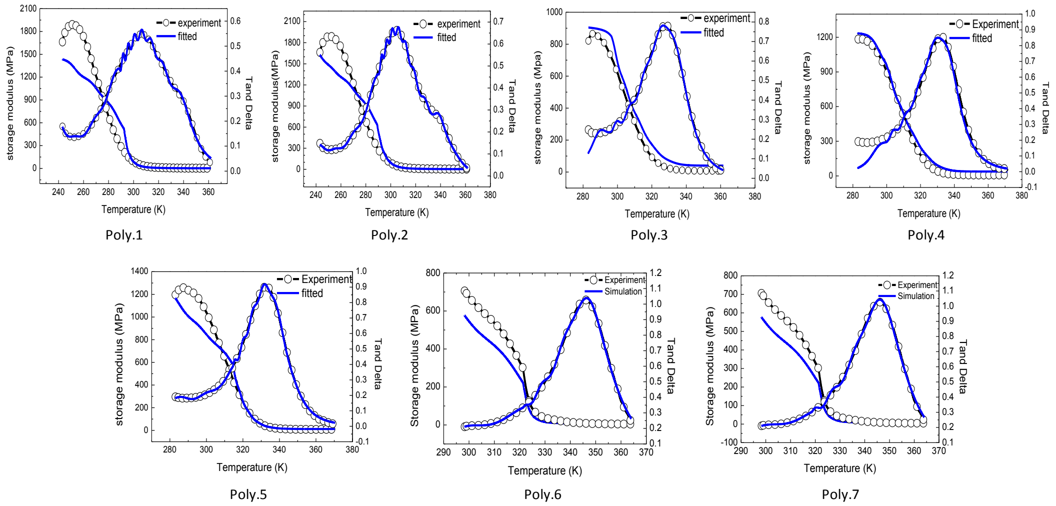

Figure S2. Fitted storage modulus and for all 7 digial materials. Blue solid lines indicate the fitted materials and black with circles indicate experiment data.

S3. Scaling rule between bending deformation and linear strain deformation

To demonstrate the scaling relation between bending deformation and linear strain deformation, we conduct FEA simulation of bending model and compare with linear strain model. In addtion, bending deformaiton can be considered as combination of linear deformation of each minor layer along thickness (as shown in Figure S3b). When considering there is no deformation of the central layer in bending process, the average linear strain change can be simplifilly defined as

$$\Delta l = l_t - l_o = \frac{\pi}{180}\left(R_t - \frac{h}{4}\right)\bar{\theta}_t - \frac{\pi}{180}\left(R_o - \frac{h}{4}\right)\bar{\theta}_o . \qquad \text{(S5)}$$

Here, R_o and R_t are the radius of the curvature of the central line at the initial and the final states; and $\bar{\theta}_o$ and $\bar{\theta}_t$ are the circular angles at the initial and final states, respectively (as shown in Figure S3a). By using relation $R_t\bar{\theta}_t = R_o\bar{\theta}_o$ (no deformation of central layer), the linear strain rate can be defined in terms of angle change rate as

$$\dot{e} = \frac{\pi h}{4}\dot{\bar{\theta}} , \text{ or } \dot{\bar{\theta}} = \frac{4}{\pi h}\dot{e} , \qquad \text{(S6)}$$

where is the thickness of the sample. The above angluar change rate relationship is applied in the following bending model. All the material parameters for the bending model are the same with that of the linear strain model.

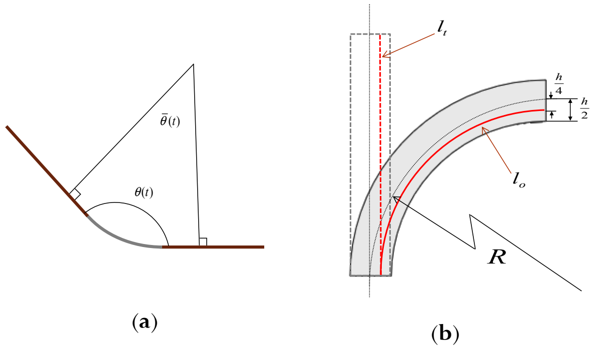

Figure S3. Schematic of hinge with thickness of h and curavature radius of R

Linear strain model: In the programming step, the SMP is stretched to a target strain e_{max} (20%) with a constant loading rate $\dot{e}$ (0.01s^{-1}) at the programming temperature T_d followed by a specified holding time at T_d before being cooled to the shape-fixing temperature T_L (10°C) at the rate of (2.5^{o}C min^{-1}). Once T_L is reached, the specimen is held for 1 hour then the tensile force is removed. Finally, the temperature is increased to the recovery temperature at the same rate of cooling and subsequently stabilized for another 50 mins. In the programming step, stress can obtained directly from Eq. S1 when the displacement controlled is used (as in our case); in the recovering step, the total strain $e(t)$ is obtained by solving Eq. S1 with a zero stress on the left-hand-side. Then the recovery ratio is obtained as

$$R_r(t) = 1 - e(t)/e_r \, , \qquad \text{(S7)}$$

where e_r is the strain measured at the start of the revovery process.

Bending model: Similar to the linear strain model, the hinge with the initial angle of $\bar{\theta}_o$=90° is programmed to a target state ($\bar{\theta}_{\max}$=0°) at the programming temperature T_d (with the maximum angle deformation of 90°), followed by a specified holding time at T_d before being cooled to the shape-fixing temperature. To have a same loading rate with that of linear strain model, the angle change rate of $\dot{\bar{\theta}} = \dfrac{0.04}{\pi h}$ is applied to arrive target deformation. The following holding condition and recover conditions are set the same with that in the linear strain model. The general shape memory cycle is shown in Figure S4a. The shape fixity is defiend as $R_f = \bar{\theta}_r / \bar{\theta}_{\max}$, and the shape recovery ratio is defined as $R_r = 1 - \bar{\theta}\,(t)/\bar{\theta}_r$. Here $\bar{\theta}_r$ is the angle at the start of the free recovery process, and $\bar{\theta}_{\max}$ is the maxiumum angle change.

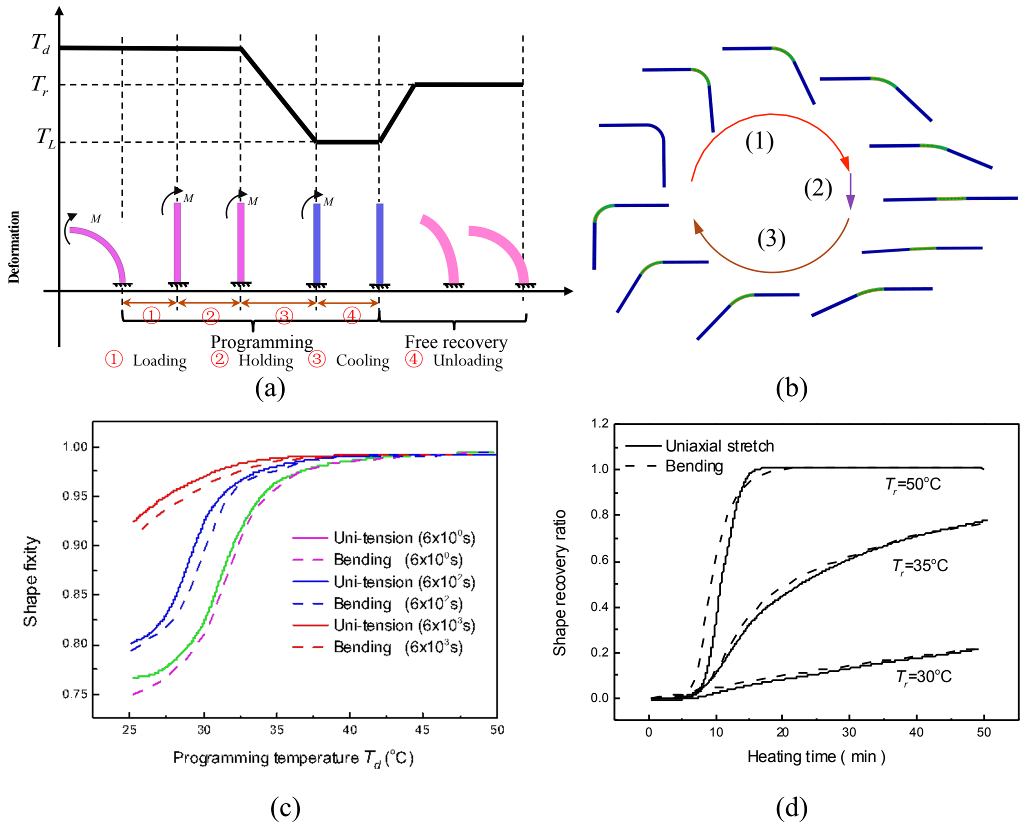

Figure S4. Shape memory simulations of SMP in bending deformation and compared with linear strain model. (a) A schematic graph of the thermomechanical history of the programming and free recovery process in an SM cycle; (b) FEA simulation on shape memory cycle of hinges; (c) shape fixity of hinge under different programming condition and compared with strain model; (d) shape recovery behaviour of hinge and compared with that by linear strain model.

From the Fig. S4c, the bending deformation has a same shape fixity with that of linear strain deformation when under same mechanical and thermal conditions. This is because the shape memory behavior is mainly affected by loading strain rate rather than the final deformation magnitude if the deformation occur in the rubbery region. Fig. S4d shows that the recovery behaviors are the same for linear strain and bending as long as they have the same fixity. It can be concluded bending deformation has a same shape memory behavior with that of linear strain

case, when the same loading strain rate as well as other mechanical and thermal conditions are applied.

References

1 Yu, K., Ge, Q. & Qi, H. J. Reduced time as a unified parameter determining fixity and free recovery of shape memory polymers. *Nat Commun* **5**, 3066, doi:10.1038/ncomms4066 (2014).

2 Aklonis, J. J. & MacKnight, W. J. *Introduction to polymer viscoelasticity*. 3rd edn, (Wiley-Interscience, 2005).

3 Christensen, R. M. *Theory of viscoelasticity*. 2nd edn, (Dover Publications, 2003).

4 Williams, M. L., Landel, R. F. & Ferry, J. D. Temperature Dependence of Relaxation Mechanisms in Amorphous Polymers and Other Glass-Forming Liquids. *Phys Rev* **98**, 1549-1549 (1955).

5 Westbrook, K. K., Kao, P. H., Castro, F., Ding, Y. & Qi, H. J. A 3D finite deformation constitutive model for amorphous shape memory polymers: A multi-branch modeling approach for nonequilibrium relaxation processes. *Mechanics of Materials* **43**, 853-869, doi:10.1016/j.mechmat.2011.09.004 (2011).
